# Depletion of dopamine in Parkinson's disease and relevant therapeutic options: A review of the literature

**DOI:** 10.3934/Neuroscience.2023017

**Published:** 2023-08-14

**Authors:** Sairam Ramesh, Arosh S. Perera Molligoda Arachchige

**Affiliations:** Department of Biomedical Sciences, Humanitas University, Milan, Italy

**Keywords:** Parkinson's disease, alpha-synuclein, dopamine, therapies, DOPAL neurotoxicity, Braak, deep brain stimulation, MAO inhibitors, COMT inhibitors, L-DOPA

## Abstract

Parkinson's disease (PD) is a progressive neurodegenerative disorder that affects motor and cognition functions. The etiology of Parkinson's disease remains largely unknown, but genetic and environmental factors are believed to play a role. The neurotransmitter dopamine is implicated in regulating movement, motivation, memory, and other physiological processes. In individuals with Parkinson's disease, the loss of dopaminergic neurons leads to a reduction in dopamine levels, which causes motor impairment and may also contribute to the cognitive deficits observed in some patients. Therefore, it is important to understand the pathophysiology that leads to the loss of dopaminergic neurons, along with reliable biomarkers that may help distinguish PD from other conditions, monitor its progression, or indicate a positive response to a therapeutic intervention. Important advances in the treatment, etiology, and pathogenesis of Parkinson's disease have been made in the past 50 years. Therefore, this review tries to explain the different possible mechanisms behind the depletion of dopamine in PD patients such as alpha-synuclein abnormalities, mitochondrial dysfunction, and 3,4-dihydroxyphenylacetaldehyde (DOPAL) toxicity, along with the current therapies we have and the ones that are in development. The clinical aspect of Parkinson's disease such as the manifestation of both motor and non-motor symptoms, and the differential diagnosis with similar neurodegenerative disease are also discussed.

## 1. Introduction

Parkinson's disease (PD) is a neurodegenerative disorder that affects millions of people worldwide. It is characterized by the progressive loss of dopamine-producing neurons in the brain, leading to a range of motor and non-motor symptoms. Parkinson's disease is the second most common neurodegenerative disorder after Alzheimer's disease, affecting approximately 1% of the population over the age of 60 worldwide. The incidence of Parkinson's disease increases with age, with a mean onset of around 60 years of age. Compared to women, men are more likely to develop Parkinson's disease, with a male-to-female ratio of approximately 1.5:1.

Parkinson's disease was first described in 1817 by British physician James Parkinson in his essay “An Essay on Shaking Palsy” [1]. In this essay, Parkinson observed six patients with symptoms such as tremors, rigidity, and difficulty with movement. He recognized that these symptoms were related to a disease of the nervous system, which he called “paralysis agitans”. Over the following decades, more and more cases of this disease were described, and the name “Parkinson's disease” became widely used. In the late 19th and early 20th centuries, researchers made important discoveries about the neuropathology of Parkinson's disease, including the loss of dopaminergic neurons in the substantia nigra region of the brain.

The incidence and prevalence of Parkinson's disease widely varies across different geographic regions and ethnic groups. Higher rates of Parkinson's disease have been reported in North America and Europe compared to Asia and Africa. The reasons for these geographic differences are not well understood, but environmental factors such as exposure to pesticides, herbicides, and other chemicals have been implicated [2]. In contrast to the general population, mortality does not increase in the first 5 years following the onset of the disease; however, it increases with a relative risk of 3.5 after 10 years, as evident from the reported data [74]. Between 2005 and 2030, it is anticipated that the number of people who have Parkinson's disease would double. Between 1990 and 2010, the number of years with a disability and disability-adjusted life years attributable to Parkinson's disease grew, and as the world's population ages, it is anticipated that the disease's financial, social, and human costs will continue to rise [3].

Apart from environmental factors, genetic factors also play a role in the development of Parkinson's disease, with several genes implicated in its pathogenesis. For example, mutations in the *SNCA, LRRK2*, and *GBA* genes have been shown to increase the risk of Parkinson's disease. Genetic studies of rare familial cases of PD led to the discovery of monogenic forms of PD, with the first PD-associated gene, *SNCA* (alpha-synuclein). Since then, several other genes have been identified as either causative or risk factors for PD, including *LRRK2, Parkin, PINK1, DJ-1*, and *VPS35*, among others [4]. These genes are involved in various cellular processes, including protein degradation, mitochondrial function, and autophagy, among others. The identification of these genes has provided valuable insights into the underlying mechanisms of PD and has helped to refine our understanding of the disease. For example, mutations in *LRRK2* have been linked to abnormalities in protein degradation pathways and have been shown to increase the risk of developing PD in various populations. Similarly, mutations in *PINK1* and *Parkin* have been linked to mitochondrial dysfunction, suggesting that defects in energy metabolism may play a role in the development of PD. However, familial Parkinson's disease accounts for approximately 10% of all cases and is characterized by a clear inheritance pattern. These mutations lead to the loss of dopaminergic neurons in the substantia nigra, resulting in motor symptoms such as tremors, rigidity, and bradykinesia, as well as non-motor symptoms such as cognitive impairment and depression. In contrast, sporadic Parkinson's disease (SPD) accounts for the majority of cases and is not directly inherited. While SPD does not have a clear genetic cause, several risk factors have been identified, including age, sex, environmental exposures, and genetic susceptibility. Environmental factors such as the ones mentioned above have been implicated in the development of SPD, as have genetic factors such as polymorphisms in genes such as *GBA, SNCA*, and *LRRK2*, among others [5].

PD is clinically characterized by classical motor symptoms, also known as Parkinsonian syndrome. They include bradykinesia, rigidity, resting tremors, and postural instability. The clinical motor symptoms of PD can have a significant impact on the patients quality of life and can make daily activities challenging. Bradykinesia, or slowness of movement (in particular, difficulty to initiate a movement), is often one of the earliest symptoms of PD and can affect activities such as walking, writing, and performing daily tasks. Rigidity, or stiffness in the muscles, can contribute to a characteristic stooped posture and can make movement difficult and uncomfortable. Resting tremor, which typically occurs in the hands, is a hallmark feature of PD, although not all people with PD experience tremors. Postural instability, which can lead to falls and other injuries, is a common feature of advanced PD [6].

Parkinson's disease patients often display a variety of motor characteristics, which has led to efforts to categorize the disease into several subgroups. There is currently no agreement on how to classify different subtypes of Parkinson's disease, but based on clinical observations, there appear to be two primary subtypes: tremor-dominant Parkinson's disease, which is characterized by a lack of other motor symptoms, and non-tremor-dominant Parkinson's disease, which includes phenotypes such as akinetic-rigid syndrome and postural instability gait disorder. Some patients with Parkinson's disease may have either a mixed or uncertain phenotype with similar severity of multiple motor symptoms. The subtypes of Parkinson's disease differ in their course and prognosis; tremor-dominant Parkinson's disease generally progresses more slowly and causes a decreased functional disability compared to non-tremor-dominant Parkinson's disease [7].

However, there are also a variety of non-motor symptoms that can occur, which can have a significant impact on the patients quality of life. Cognitive impairment is one of the main non-motor symptoms of PD. PD can cause a range of cognitive problems, including problems with memory, attention, and executive function, among others. Studies have found that up to 80% of PD patients may experience some form of cognitive impairment during the course of the disease [8]. Mood disorders, such as depression and anxiety, are also common in PD and can have a significant impact on the patients quality of life. Depression is one of the most common non-motor symptoms in PD, with an estimated prevalence of up to 50% [9]. Anxiety is also common, with up to 30% of PD patients experiencing anxiety symptoms. PD can affect the autonomic nervous system, leading to problems with blood pressure, heart rate, digestion, and bladder and bowel function, among other issues. Autonomic dysfunction can have a significant impact on quality of life and can be a major source of disability in PD patients. Sleep disturbances are another common non-motor symptom of PD. PD can cause a variety of sleep problems, including insomnia, restless leg syndrome, and REM sleep behavior disorder. Sleep disturbances can have a significant impact on the patients quality of life and can also contribute to cognitive impairment and mood disorders. Moreover, PD can cause a variety of sensory symptoms, including a loss of sense of smell, pain, and vision problems. Sensory symptoms can be distressing and can further impact the quality of life in PD patients. It is common for non-motor symptoms (in particular olfactory ones) to be present in Parkinson's disease, even before the typical motor symptoms appear [10].

The development of Parkinson's disease is believed to begin during the premotor phase, affecting various regions of both the peripheral and central nervous systems, as well as the dopaminergic neurons located in the substantia nigra pars compacta [11]. Moreover, these premotor markers may be used to detect the development of Parkinson's disease before the motor problems manifest.

Among the several factors that play a role in the progression of motor symptoms in PD, dopamine remains the crucial one. The role of dopamine in the progression of PD is quite complex and not fully understood. Parkinson's disease is characterized by a reduction in dopamine levels due to the loss of dopaminergic neurons in the substantia nigra, resulting in an imbalance between the direct and indirect pathways in the striatum. This imbalance causes overactivity of the indirect pathway and a reduction in the activity of the direct pathway, leading to typical motor symptoms such as tremors, rigidity, and bradykinesia. This was first discovered in a landmark paper published by Dr. Oleh Hornykiewicz, who demonstrated a considerable reduction of dopamine levels in the caudate and putamen of Parkinson's and post-encephalitic Parkinsonian brains. Following several additional papers on the post-mortem biochemistry of Parkinson's disease, Hornykiewicz published a key review article in Pharmacological Reviews, proposing that the deficiency of striatal dopamine is correlated with the majority of Parkinson's disease's motor symptoms [12]. To develop effective treatments for Parkinson's disease, it is essential to understand the role of dopamine in both motor control and other cognitive and emotional processes.

## 2. Pathophysiology

The basal ganglia is a complex group of subcortical nuclei located in the deep parts of the brain and plays a vital role in regulating various functions, including movement, cognition, and emotion. It consists of various nuclei, namely the caudate nucleus, putamen, globus pallidus, subthalamic nucleus, and substantia nigra, each with their specific functions. The circuits within the basal ganglia are intricate and involve various pathways that influence the activity of both cortical and subcortical structures [13].

The two distinct pathways of the basal ganglia include the direct and indirect circuits that are involved in the regulation of movement. These pathways originate from the striatum, which is composed of the putamen and caudate nucleus, and target different nuclei within the basal ganglia.

**Figure 1.**
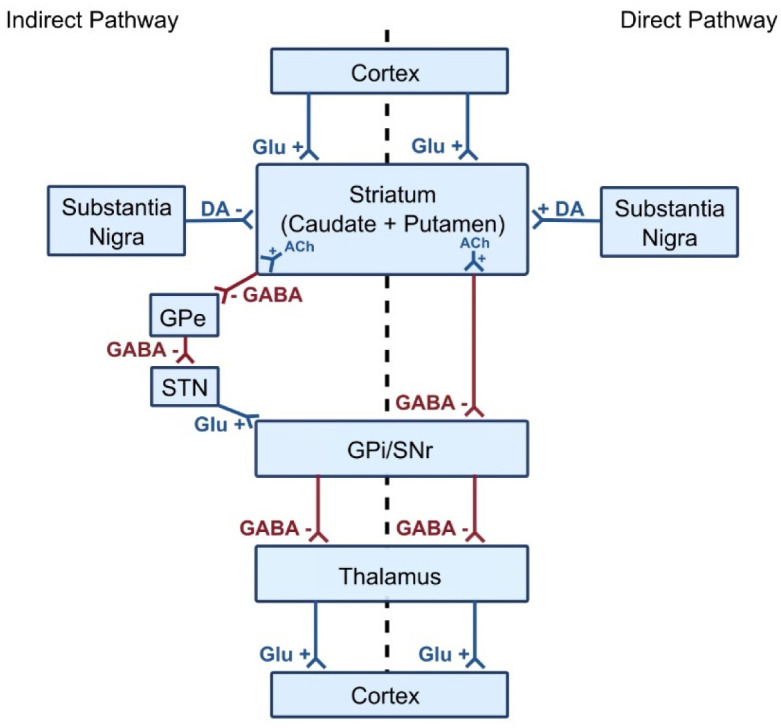
Diagram illustrating the direct and indirect pathways of the basal ganglia, which play a critical role in motor control. The direct pathway facilitates desired motor actions by reducing inhibition of the thalamus, promoting movement. In contrast, the indirect pathway suppresses unwanted movements by increasing thalamic inhibition. Together, they fine-tune motor movements, ensuring smooth and coordinated actions.

The direct pathway promotes movement by facilitating (dis-inhibiting) thalamic activity. It is made up of GABA-ergic medium spiny neurons (MSN) that also express dynorphin, substance P, and D1 receptors and directly project to the internal segment of the globus pallidus (GPi). When D1 receptors on MSNs are activated, the protein kinase A (PKA) signaling pathway is activated, leading to the inhibition of the inhibitory neurons in the GPi. This subsequently reduces the inhibition of the thalamus, which leads to an increase in cortical activity and facilitates movement. The indirect pathway is involved in inhibiting movement by increasing the inhibitory output to the thalamus. It is made up of GABA-ergic medium spiny neurons (MSN), though they express enkephalin, neurotensin, and D2 receptors; these neurons project to the external segment of the globus pallidus (GPe). Activation of D2 receptors on MSNs leads to a decrease in cAMP levels and a reduced PKA activity due to the inhibition of the adenylyl cyclase signaling pathway. The decreased activity of these MSN leads to the disinhibition of inhibitory neurons in the GPe that subsequently inhibits the subthalamic nucleus (STN). The inhibition of the STN results in the decreased activation of the GPi, leading to the decreased inhibition of the thalamus and of cortical activity, again facilitating movement [13]. See Figure 1.

At its core, Parkinson's disease is characterized by the loss of dopaminergic neurons in the substantia nigra pars compacta (SNpc), most notably in the ventrolateral tier, where the neurons that project to the dorsal putamen of the striatum are present. Dopamine is synthesized in the cytosol and is then transported into synaptic vesicles via the vesicular monoamine transporter 2 (VMAT2). Enzymes involved in the synthesis of catecholamines are transported from the cell body in the substantia nigra to nerve terminals, where dopamine is formed. After its release, dopamine can interact with both pre- and postsynaptic dopamine receptors. When the transport of dopamine into synaptic vesicles is obstructed or VMAT2 is inhibited by drugs such as tetrabenazine and reserpine, excessive dopamine can accumulate in the cytoplasm, leading to the auto-oxidation of dopamine and the formation of harmful by-products, eventually leading to neurodegeneration. To protect against this, neurons have defense mechanisms that involve either condensing cytosolic dopamine into neuromelanin or breaking down dopamine via MAO and COMT to generate homovanillic acid. Due to this metabolic pathway, treatment with reserpine or tetrabenazine leads to the depletion of brain catecholamines [14].

However, the loss of dopaminergic neurons is not the only characteristic feature of PD, with widespread the intracellular accumulation of alpha-synuclein being a key feature. Alpha-synuclein (α-syn) is a small, intrinsically disordered protein that is predominantly found in the presynaptic terminals of neurons. It is involved in synaptic vesicle trafficking and regulation of neurotransmitter release. When α-synuclein loses its proper folding, it becomes unable to dissolve and instead forms inclusions inside neurons' cell bodies and processes. These inclusions, called Lewy bodies and Lewy neurites, are typical features of Lewy pathology (LP) in Parkinson's disease. Not only are they found in the brain, but they can also be present in the spinal cord and other parts of the nervous system, including the vagus nerve, sympathetic ganglia, cardiac plexus, enteric nervous system, salivary glands, adrenal medulla, cutaneous nerves, and sciatic nerve [15]. These α-syn aggregates are thought to play a central role in the pathogenesis of PD. The body maintains a balance of α-synuclein levels within cells through two cellular degradation systems: the ubiquitin-proteasome system and the lysosomal autophagy system (LAS). Recent studies have suggested that the LAS is more important than the ubiquitin-proteasome system in clearing oligomeric assemblies of α-synuclein. The two mechanisms within LAS, chaperone-mediated autophagy, and macroautophagy, are both suggested to play a role in α-synuclein degradation. Inhibition of either system leads to increased levels of α-synuclein, and some evidence suggests that there is compensatory crosstalk between the two systems. Additionally, there are other proteases in the extracellular space that can cleave α-synuclein. Impairment of these degradation systems is thought to contribute to the accumulation of α-synuclein in the body [16]. Although the loss of pigmented dopaminergic neurons in the substantia nigra and the deposition of α-synuclein in neurons are not exclusive to Parkinson's disease, their combination is necessary for a confirmed diagnosis of idiopathic Parkinson's disease [17].

**Figure 2.**
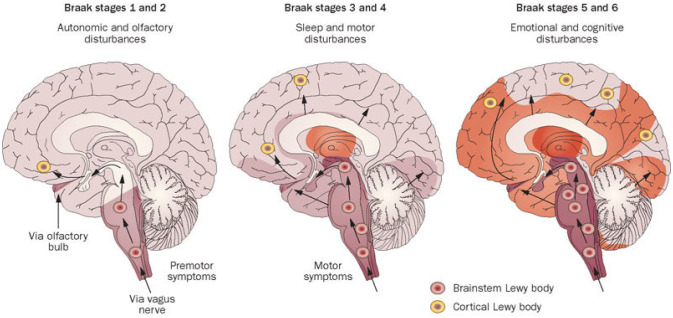
Braak staging is used to classify the degree of pathology in PD [18].

Researchers have proposed that Lewy pathology, the aggregation of α-synuclein in the brain, progresses in a specific pattern in Parkinson's disease. As proposed by Braak and colleagues, this pattern involves six stages, starting in the peripheral nervous system and gradually affecting the central nervous system in a caudal-to-rostral direction within the brain. In individuals without any symptoms of Parkinson's disease, α-synuclein inclusions can be found in the lower brainstem neurons that are involved in the cholinergic and monoaminergic systems (Braak stage I and stage II). As the disease progresses and motor symptoms develop, similar neurons in the midbrain and basal forebrain become infiltrated with α-synuclein inclusions (Braak stage III and stage IV). With further disease progression, these inclusions are then found in regions of the brain involved in limbic and neocortical function (Braak stage V and stage VI) [18]. See Figure 2.

The Braak model is gaining support because it appears to explain the clinical course of Parkinson's disease. The first two stages may correspond to the onset of premotor symptoms, while stage 3 is when motor features emerge due to dopamine deficiency in the nigrostriatal pathway. Stages 4–6 may occur alongside the non-motor symptoms of advanced disease. There is strong evidence that Lewy pathology is associated with cognitive impairment in Parkinson's disease [19].

A new theory for the formation of α-synuclein aggregates has emerged, known as the prion-like hypothesis. According to this hypothesis, once α-synuclein aggregates form within a neuron, they can be transported to other regions of the brain through axons, released into the extracellular space, and taken up by nearby neurons. Once inside the new host neuron, the aggregates can then seed the aggregation of endogenous α-synuclein, leading to the formation of more aggregates. At the onset, the aggregation of α-synuclein in a few cells could result in the gradual transmission of these aggregates to other brain regions over a period of years or decades [20].

According to several neuropathological reports, it has to be noted that clinical PD can also manifest without any Lewy pathology. There have been several documented cases of patients with Parkinson's disease in the absence of Lewy pathology, who have parkin-related disease and in some cases a mutation in the *LRRK2* gene.

Mutations in several genes have been associated with monogenic forms of Parkinson's disease, including *SNCA, LRRK2, Parkin, PINK1*, and *DJ-1*. These genes are involved in multiple cellular processes, such as alpha-synuclein regulation, protein degradation, and mitochondrial function, that are essential for maintaining proper neuronal function. Mutations in these genes can lead to abnormal protein build-up, oxidative stress, and mitochondrial dysfunction, all of which contribute to Parkinson's disease pathogenesis and neuronal death. Along with monogenic forms, studies have also revealed the involvement of common genetic variants in Parkinson's disease development, present in several genes with a small individual effect but the cumulative impact in increasing the disease risk.

Variants in genes responsible for dopamine metabolism, synaptic function, and neuroinflammation have been identified as risk factors for Parkinson's disease. Additionally, genetics can also influence Parkinson's disease's age of onset and clinical phenotype. For instance, *LRRK2* gene mutations increase the risk of developing Parkinson's disease with a late onset and less severe phenotype, whereas *PARK2* gene mutations are associated with early onset and a more severe phenotype [21].

Neuroinflammation is believed to be an important factor in the pathogenesis of Parkinson's disease. While inflammation is a normal immune response to either infection or injury, chronic and sustained inflammation can lead to damage to cells and tissues. Parkinson's disease is characterized by the accumulation of misfolded proteins, such as alpha-synuclein, which can activate microglia and cause them to release pro-inflammatory molecules that can harm neurons. Furthermore, neuroinflammation may contribute to the progression of Parkinson's disease by facilitating the spread of alpha-synuclein aggregates between neurons. A meta-analysis of genome-wide association data has revealed that there is a single-nucleotide polymorphism in the human leukocyte antigen (HLA) region that may influence the risk of developing Parkinson's disease. This suggests that there may be a genetic susceptibility to Parkinson's disease that is related to the immune system [22].

Anti-inflammatory drugs, such as nonsteroidal anti-inflammatory drugs (NSAIDs) and minocycline, have been studied for their potential to slow the progression of Parkinson's disease. The use of calcium channel blockers and high levels of serum urate have also been linked to a decreased risk of developing Parkinson's disease. One possible explanation for the association between the use of calcium channel blockers and elevated concentrations of serum urate and a reduced risk of Parkinson's disease is their ability to reduce oxidative stress in neurons that are vulnerable to degeneration in Parkinson's disease. Calcium channel blockers may protect against oxidative stress by reducing calcium influx into neurons, which can lead to the generation of free radicals and subsequent damage to cellular structures. Similarly, urate has antioxidant properties and can scavenge free radicals, thereby reducing oxidative stress in cells. The reduction of oxidative stress by these agents may protect against neuronal death and slow the progression of Parkinson's disease [23].

Studies have provided evidence that substantia nigra pars compacta (SNpc) neurons are highly susceptible to cell death in Parkinson's disease due to oxidative stress. This is because these neurons have high levels of mitochondrial activity, which leads to the generation of reactive oxygen species (ROS) as a by-product. The redox activity of dopamine itself is a key contributor to oxidative stress. In addition, dopamine can react with other molecules such as cysteine and glutathione, this results in a state of oxidative stress that can damage cellular components and eventually cause cell death. Oxidative stress has been shown to contribute to the accumulation of α-synuclein and the formation of Lewy bodies, which are characteristic pathological features of the disease.

Chronic oxidative stress can trigger the activation of microglia, leading to the release of pro-inflammatory cytokines and other molecules that can cause damage to neurons and promote neuroinflammation. This vicious cycle of oxidative stress and neuroinflammation can contribute to the progression of neurodegenerative diseases such as Parkinson's disease [24].

## 3. Dopamine and Parkinson's disease

The reason why dopaminergic neurons of SNpc are more susceptible to damage in Parkinson's disease is still unknown and remains a major topic of research in the field. The loss of SNpc dopaminergic (DA) neurons causes bradykinesia and stiffness, the two primary motor symptoms of PD. There have been several suggested phenotypic traits that may contribute to the vulnerability of dopaminergic neurons in the SNpc. This review briefly concentrates on current thinking on the susceptibility of this specific group of neurons, despite the fact that it is widely acknowledged that the pathology in PD is not solely restricted to SNpc DA neurons.

**Figure 3.**
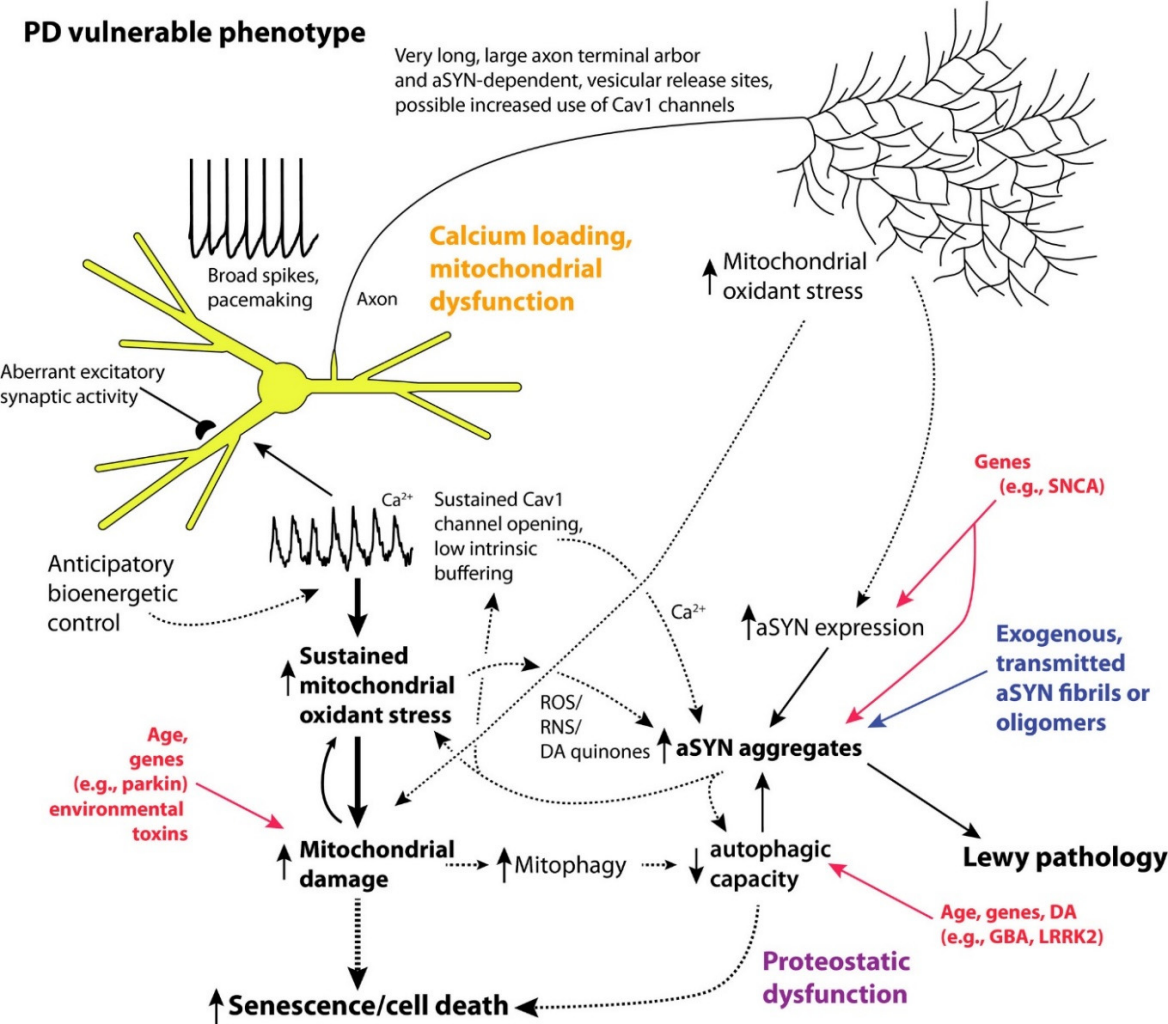
Illustrative representation of key traits of vulnerable neurons in PD. Concisely, neurons susceptible to Parkinson's disease (PD) exhibit key traits, and the disease is primarily driven by a malfunction in mitochondria and proteostasis. Mitochondrial dysfunction may result from the regulation of mitochondrial respiration by calcium and unidentified axonal bioenergetic factors, compounded by genetic and environmental influences (e.g., toxins). Proteostatic dysfunction arises from aSYN aggregation, promoted by oxidant stress, elevated cytosolic calcium, and DA quinones, as well as lysosomal dysfunction induced by increased mitophagy and oxidant damage to lysosomal proteins such as glucocerebrosidase. Solid lines represent firmly established connections in mammalian models, while dashed lines indicate mechanisms with strong but not definitive support [25].

To explain why dopaminergic neurons in the substantia nigra pars compacta (SNpc) are destroyed in Parkinson's disease, there are two primary explanations. One explanation is based on the finding that the SNpc of PD patients frequently has Lewy pathology, which includes protein aggregates rich in the fibrillary forms of alpha-synuclein (aSYN). See Figure 3. It is interesting that nearly all neurons that experience cell death in PD also express LP, even with the caveat that immunolabels for aSYN aggregates are also associated with other illnesses that sporadically overlap with PD. Contrarily, several peripheral and central neurons are observed to express LP without any indication of impending neuronal death, suggesting that the opposite is not the case [26]. How protein aggregates, such as Lewy pathology or oligomeric aSYN, develop in a small number of neurons in the mesencephalon is a critical subject in Parkinson's disease research. According to evidence from post-mortem studies on patients with Parkinson's disease, synaptically coupled networks allow LP to spread from either the olfactory bulb or the dorsal motor nucleus of the vagus (DMV) in the caudal medulla to the substantia nigra pars compacta (SNpc) during the preclinical stages of the disease. In one experiment, fetal transplants into the striatum of PD patients showed protein inclusions that closely mirrored LP, demonstrating that aSYN pathology moved from the host into the graft [27]. These experiments have demonstrated that aSYN disease can spread. When synthetic aSYN fibrils were injected into mice's brains, they caused Lewy-like disease in surrounding areas. Similar to this, proteins from human brains that were isolated by LP multiplied in monkeys [27]. In recent investigations, surface proteins that particularly interact with aSYN fibrils and encourage their spreading have been discovered. These experiments show that extracellular aSYN aggregates can be absorbed, disseminate, and cause LP despite their limitations [28].

**Figure 4.**
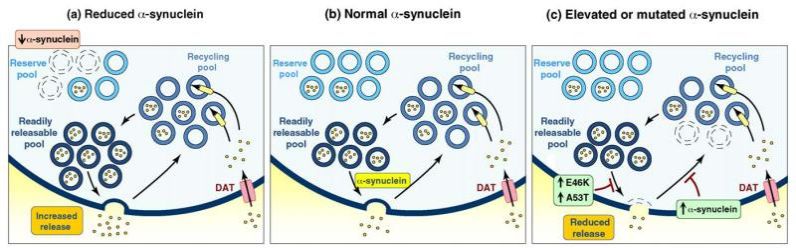
Diagram showing the proposed functions of α-synuclein in controlling the cycling of presynaptic vesicles under varying levels of α-synuclein: (a) When α-synuclein levels are decreased, the reserve pool of vesicles is reduced, resulting in a higher number of readily available vesicles for release. This could lead to an augmentation in dopamine release. (b) In normal conditions, α-synuclein is believed to have a physiological role in regulating vesicle availability across different pools and in vesicle docking and fusion. (c) Conversely, elevated α-synuclein levels or mutations like *E46K* or *A53T* α-synuclein lead to a decrease in dopamine release. This may be due to their potential impact on a late stage in exocytosis or by reducing vesicle availability in the recycling pool through impaired vesicle endocytosis [32].

The relationship between aSYN pathology, cell death, and symptoms in PD remains uncertain, despite the clear evidence of widespread aSYN-laden LP in the disease and the ability of aSYN aggregates to spread in animal models. One particularly intriguing question is why SNpc DA neurons should be particularly susceptible to propagated aSYN aggregates. Although studies have shown that aSYN fibrils injected into the brain can cause neuronal death, LP does not appear to be particularly toxic at lower, more biologically relevant concentrations. In fact, LP can persist in many parts of the brain, including the brainstem, for decades without causing any observable degeneration or cell death. It is unclear why DMV neurons can tolerate LP, while SNpc DA neurons cannot. In both mouse and cell culture models, the analysis of non-dopaminergic hippocampal neurons provided some evidence for a link between aSYN and synaptic vesicle function. Mice that lack the expression of aSYN exhibited dysfunction in the ability of hippocampal synapses to respond to prolonged stimulation, which is expected to cause depletion of the docked and reserve pool of synaptic vesicles. In addition, there is a reduction in the replenishment of docked pools from the reserve pool [29]. These findings are consistent with the idea that α-synuclein plays a crucial role in the regulation of synaptic vesicles at presynaptic terminals. This is further supported by the observation that suppression of α-synuclein, achieved using antisense oligonucleotides in primary cultured hippocampal neurons, decreases the availability of a “distal” or reserve synaptic vesicle pool. On the other hand, studies have found that transgenic mice overexpressing human aSYN exhibit impaired synaptic vesicle exocytosis in their hippocampal neurons [30]. Similarly, when rat ventral midbrain dopaminergic neurons were transfected with aSYN, there was also a reduction in synaptic vesicle exocytosis. The overexpression of aSYN in hippocampal neurons led to a decrease in the size of the synaptic vesicle recycling pool, as it affected the reclustering of synaptic vesicles following endocytosis [31]. According to these studies on dopaminergic systems, synuclein removal causes an increase in dopamine release, whereas aSYN overexpression causes a decrease in dopamine release. These results imply that aSYN might influence vesicle fusion during synaptic stimulation and the distribution of vesicles between the ready releasable and reserve synaptic pools, acting as a negative regulator of dopamine release. However, to fully understand how aSYN affects dopaminergic vesicles to control dopamine release, more research is necessary. See Figure 4. It is also worth noting that SNpc DA neurons appear to be lost in sporadic PD cases before LP is present in the SNpc, and LP is not present in some familial cases despite the loss of SNpc DA neurons, thus being a criticism of this hypothesis. This is further supported by the understanding that one form of familial Parkinson's disease (PARK4) is due to the overexpression of aSYN, which is likely to favor the misfolding, aggregation and precipitation of the protein.

The second hypothesis (which is not mutually exclusive) is that mitochondrial malfunction is what causes the loss of SNpc DA neurons in PD. Studies of familial cases of PD provide a significant amount of support for this assertion. *Parkin (PARK 2), PINK1 (PARK 6)*, and *DJ-1 (PARK 7)* loss-of-function mutations result in recessive, early-onset variants of Parkinson's disease (PD). Additionally, the three gene products have direct effects on mitochondrial biology, regulating a variety of processes including oxidative phosphorylation, quality control, and oxidant defenses [33].

Mitochondria have been linked to pathogenesis in PD patients' brains after several post-mortem analysis. In the SNpc of PD patients, functional complex I levels are reduced. Due to the fact that functional complex I levels are lower in surviving SNpc DA neurons, this is not merely a result of neurodegeneration. The mitochondrial electron transport chain (ETC), more specfically mitochondrial complex I, is also invariably inhibited by toxins associated with Parkinson's disease (PD), such as rotenone [34].

However, this leads us to the question of why should SNpc DA neurons be particularly vulnerable to mitochondrial dysfunction more than any aSYN pathology.

It has been proposed that SNpc DA neurons have three properties that make them more susceptible to these assaults. One distinguishing feature of SNpc neurons is their abnormally long, branching axons that are either unmyelinated or thinly myelinated. One SNpc axon ending in the striatum can have up to several hundred thousand synaptic release sites and is extremely branched. This exceeds the majority of extensively examined neurons by an order of magnitude or more. There is currently no evidence suggesting that there is an elevated level of oxidative stress in the mitochondria of these terminals. However, maintaining a large terminal field would probably impose a significant metabolic and proteostatic burden on the cell body. Given the low density of mitochondria in the somatodendritic region of SNpc DA neurons, the transportation of mitochondria to axons may be necessary, which presents a challenge for mitochondrial trafficking [35]. It is also quite likely that the incredibly vast axonal arbour of SNpc DA neurons will boost the expression of aSYN (which is largely a synaptic protein).

Another distinctive feature of SNpc DA neurons is their unique physiology. These neurons produce broad, sluggish action potentials that promote slow rhythmic activity and boost calcium influx. Slow oscillations in the intracellular calcium concentration are accompanied by this autonomous activity, which has a frequency range of 2 to 10 Hz. These calcium oscillations are initiated by the activation of plasma membrane Cav1 calcium channels and the release of calcium from intracellular ER reserves. Because calcium buffering proteins like calbindin are scarce in the cytoplasm, calcium instead interacts with other proteins there. The characteristics of SNpc DA neurons set them apart from the majority of brain neurons, including large spikes, delayed pacemaking, low intrinsic calcium buffering, and cytosolic calcium oscillations. Conversely, VTA DA neurons, which are less vulnerable than SNpc DA neurons, generate broad spikes and are autonomous pacemakers, but have smaller Cav1 channel currents and a strong intrinsic calcium buffering by calbindin. [36] The slow calcium oscillations in SNpc DA neurons serve two functions that work together. First, they help to maintain the slow, rhythmic firing pattern by generating an oscillation in the membrane potential. Second, they promote calcium to enter mitochondria through specialized junctions between the mitochondria and the endoplasmic reticulum. However, this leads to stimulating oxidative phosphorylation, which, in the absence of strong ATP demand, leads to mitochondrial hyperpolarization, that eventually leads to an increase in ROS and RNS, both of which can damage the mitochondria. This may be a significant contributing component to the aging-related decline in mitochondrial function in vulnerable neurons. Moreover, ROS and RNS increase the propensity of aSYN to aggregate and worsen the effects of genetic mutations and environmental pollutants on the mitochondria [37].

Therefore, SNpc DA neurons are located near critical points in bioenergetic and protein degradation processes. As age increases, mitochondrial and proteasomal/autophagic functions are affected, which is the biggest risk factor for PD. This will undoubtedly bring them closer to the tipping point, increasing the likelihood of either developing LP or being unable to manage the burden caused by taking up pathological aSYN species from the extracellular space.

## 4. DOPAL Neurotoxicity

Amongst the several determinants of the degeneration of SNpc DA neurons, the possible endotoxicity associated to dopamine dyshomeostasis is an important factor to consider. Particularly, the relevance of reactive dopamine metabolite 3,4-dihydroxyphenylacetaldehyde (DOPAL) in catechol-induced neurotoxicity. The combined effect of the catechol and aldehyde components in DOPAL increases its reactivity, leading to the modification of functional protein residues, protein aggregation, oxidative stress, and cell death. Notably, DOPAL is known to preferentially target α-synuclein, which is a key factor in Parkinson's Disease pathology due to altered proteostasis. DOPAL triggers the oligomerization of αSynuclein, which impairs synapse physiology.

Within SNpc neurons, the levels of dopamine are carefully regulated through a balance between synthesis, synaptic vesicle loading, uptake from the extracellular space, and catabolic degradation. The catabolism of dopamine begins with oxidative deamination, which is mediated by mitochondrial monoamine oxidase (MAO) and generates H_2_O_2_ and ammonia. The resulting product, DOPAL, is further metabolized to either 3,4-dihydroxyphenylacetic acid (DOPAC) or 3,4-dihydroxyphenylethanol (DOPET) by either aldehyde dehydrogenase (ALDH) or by aldehyde/aldose reductase (ALR/AR), respectively.

**Figure 5.**
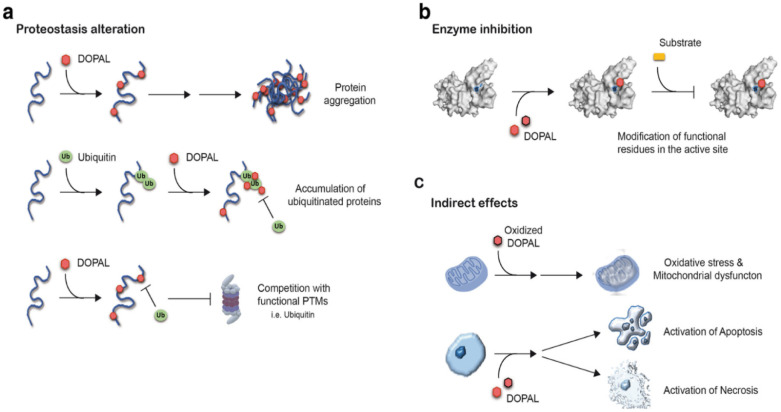
Neurotoxic molecular mechanisms associated with DOPAL (dihydroxyphenylacetaldehyde): DOPAL accumulation in dopaminergic neurons of the substantia nigra pars compacta (SNpc) triggers several neurotoxic processes: (a) Disruption of neuronal proteostasis, leading to protein aggregation, competition with functional post-translational modifications (PTMs) such as ubiquitination, SUMOylation, and acetylation, and buildup of ubiquitinated proteins. (b) Inhibition of enzymes within the neurons. (c) Indirect effects, including oxidative stress, mitochondrial dysfunction, and activation of necrotic and apoptotic pathways [39].

While DOPAL is a natural intermediate in the catabolism of dopamine, it has been shown to have neurotoxic effects. See Figure 5 [38]. In various cell lines, concentrations of DOPAL higher than the physiological range (>6 µM) have been reported to be cytotoxic. There are several reasons why DOPAL can accumulate at pre-synaptic terminals. These include dopamine leakage from synaptic vesicles, an increased rate of dopamine conversion to DOPAL due to upregulated monoamine oxidase, and a decrease in DOPAL degradation caused by aldehyde dehydrogenases. DOPAL has two functional groups, the aldehyde and catechol moieties, that contribute to its high reactivity and toxicity. The aldehyde group mainly targets primary amines, while the catechol group targets thiols in proteins. A study conducted post-mortem on the brains of sporadic Parkinson's disease patients showed an accumulation of DOPAL compared to DA in the putamen region of healthy individuals. The results of the study lead to the formulation of the catecholaldehyde hypothesis, which proposes that the accumulation of DOPAL, a reactive metabolite of dopamine, plays a central role in the degeneration of SNpc neurons in Parkinson's disease.

Monoamine oxidase inhibitor (MAO) inhibitors have been used as a treatment for Parkinson's disease for many years and have FDA approval. The MAO inhibition strategy appears even more promising in light of the catecholaldehyde hypothesis because it can obstruct one of the causes of DOPAL accumulation. A 2016 study by Goldstein et al. examined the effectiveness of different MAO-A and MAO-B inhibitors in lowering DOPAL levels in PC-12 cells. The findings provided a proof of concept for this therapeutic strategy by demonstrating the efficiency of clorgyline, rasagiline, and selegiline in inhibiting MAO and decreasing endogenous DOPAL synthesis [40]. It is crucial to take the potential drawbacks of MAO inhibition into account because it can raise cytosolic dopamine levels, which can reduce TH activity through feedback inhibition. Moreover, dopamine may experience auto-oxidation and result in oxidative stress if it is not correctly stored in synaptic vesicles. In this case, the neurotoxicity of cytosolic dopamine would prevent the benefits of lowering DOPAL levels [40].

However, a deeper comprehension of the DA catabolic pathway and how it functions in PD patients will enable the development of more individualized and efficient therapy approaches.

## 5. Clinical considerations

Studies have shown that neuronal loss in the SNpc begins around five years before diagnosis, while clinical studies have estimated six to seven years pre-diagnosis. More recent studies using VMAT2 and DA transporter radiotracers, which are more sensitive to early abnormalities than ^18^F-DOPA PET, have estimated the initial decline in dopaminergic pre-synaptic activity to be up to 17 years before diagnosis. It can be concluded that the dopaminergic deficit is efficiently compensated for a long period of time, at least 10 years or longer, during the normal or near-normal motor activity (silent/prodromal motor period) before clinical symptoms are sufficient for diagnosis (manifested period) [41].

During the silent motor period, from a clinical point of view, there are no observable motor symptoms. The prodromal motor period follows, during which, mild motor symptoms first become noticeable. The duration of the prodromal motor period varies among individuals and is influenced by the initial manifestation of motor symptoms. In most cases, the upper limb and hand are usually the first affected [42]. The clinical signs of Parkinson's disease, including motor symptoms, can be easily overlooked or attributed to other causes, leading to delays in diagnoses. In the early stages, fine hand movements may slow down, arm swings may decrease, gait may change, or there may be subtle tremors. These signs are often not specific to Parkinson's disease and can be mistaken for other conditions, contributing to delays in diagnoses.

PD usually begins with unilateral motor symptoms, and this asymmetry persists throughout the disease course. The average age of onset is in the late 50s, although it can range from younger than 40 years to older than 80 years. When Parkinson's disease begins before the age of 45 years, it is known as early-onset Parkinson's disease. More than 10% of these cases have a genetic basis, and the proportion of genetically defined cases increases to over 40% among those with a disease onset before the age of 30 years [43]. Non-motor symptoms in Parkinson's disease encompass a variety of functions, such as disturbances in sleep-wake cycle regulation, cognitive dysfunction (such as frontal executive dysfunction, memory retrieval problems, dementia, and hallucinations), mood and affect disorders, autonomic dysfunction (primarily orthostatic hypotension, urogenital dysfunction, constipation, and excessive sweating), sensory symptoms (especially reduced sense of smell), and pain. As the illness progresses, non-motor symptoms become more common and have a significant impact on the quality of life, overall disability, and the need for nursing home care.

## 6. Diagnosis

The clinical diagnosis of Parkinson's disease relies on the identification of motor symptoms such as bradykinesia, rigidity, and resting tremor. Diagnosing Parkinson's disease in its early stages can be challenging, with error rates as high as 24%, even in specialized medical centers. While using standard clinical criteria such as the UK Parkinson's Disease Society Brain Bank criteria can improve accuracy, it still only reaches slightly above 80% accuracy during the first visit. A recent meta-analysis of 11 studies that compared clinical diagnoses based on UKPDSBB criteria with post-mortem pathological examination as the gold standard showed only slightly improved accuracy. However, in cases where classic motor features of Parkinson's disease are fully developed, a diagnosis can be more straightforward [44]. Even before the beginning of motor symptoms, strategies to identify biomarkers for the diagnosis of Parkinson's disease are being researched, specifically aiming to enable detection early in the disease's course. Although Parkinson's disease-modifying medications are not yet available, it is predicted that they will be most effective if patients can be identified and treated at this prodromal premotor stage. The University of Pennsylvania's smell identification test and the polysomnography-detected REM sleep behavior disorder are two examples of potential clinical markers. Olfactory impairment can also be detected using established techniques [45].

The prodromal stage in Parkinson's disease refers to the period before the onset of classic motor symptoms when non-motor symptoms may be present. These non-motor symptoms can include olfactory impairment, sleep disturbances, constipation, mood changes, and other subtle changes that can occur years before the appearance of motor symptoms. Identifying biomarkers during this prodromal stage is crucial for early detection and intervention. One promising area of research involves the use of α-synuclein seed amplification assays in either the blood or cerebrospinal fluid. α-synuclein is a protein that plays a central role in the pathogenesis of Parkinson's disease, and abnormal aggregation of this protein is a key feature of the condition. The α-synuclein seed amplification assays aim to detect these abnormal protein aggregates even before motor symptoms manifest [71]. These assays are being studied as potential biomarkers for early diagnosis and monitoring of disease progression. By identifying α-synuclein aggregates in the prodromal stage, it may be possible to intervene with disease-modifying therapies earlier, potentially slowing down or preventing the neurodegenerative process. Including information about these new approaches, such as α-synuclein seed amplification assays in either the blood or CSF, would enhance the diagnostic section and highlight the cutting-edge research being conducted to improve the early detection and management of Parkinson's disease [72].

### 6.1. Imaging

**Figure 6.**
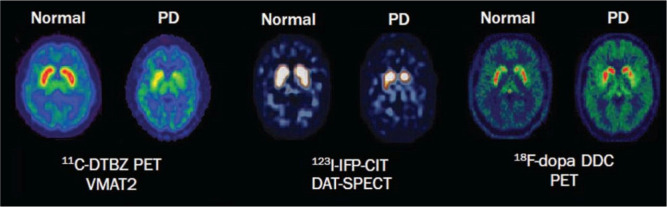
Molecular imaging of dopaminergic dysfunction in Parkinson's disease (PD) involves the use of PET (positron emission tomography) and SPECT (single-photon emission computed tomography) imaging techniques. In PD patients, these imaging studies reveal a reduction in VMAT2 (type 2 vesicular monoamine transporter) activity, DAT (dopamine transporter) availability, and DDC (dopa decarboxylase) activity when compared to healthy individuals used as controls. PET and SPECT imaging provides valuable insights into the molecular changes associated with PD, showcasing lower levels of VMAT2, DAT, and DDC functions, which are indicative of dopaminergic dysfunction in the disease [48].

The visualization of striatal dopamine depletion using ^18^F-labelled L-DOPA and PET in patients with Parkinson's disease was a significant advancement in molecular neuroimaging in the 1980s. This allowed for the detection and monitoring of dopamine deficiency in the brain, which is a hallmark feature of Parkinson's disease. See Figure 6 [46].

However, over the years, technological advancements in medical imaging have enabled the detection of structural changes in the substantia nigra in Parkinson's disease patients using both transcranial sonography and diffusion tensor magnetic resonance imaging (MRI). While transcranial sonography can identify susceptibility to PD, PET and SPECT measurements of dopamine terminal function can detect dopamine deficiency in both symptomatic and at-risk subjects for Parkinsonian syndromes. Moreover, measurements of striatal dopamine deficiency can provide biomarkers for monitoring disease progression and correlate with bradykinesia and rigidity. Conversely, normal striatal DAT binding is associated with a good prognosis and excludes a dopamine deficiency syndrome in a suspected PD patient. In addition, diffusion-weighted MRI and ^18^F-fluorodeoxyglucose positron emission tomography (^18^F-FDG PET) can be used to distinguish atypical Parkinsonian syndromes from PD with high sensitivity [47].

### 6.2. Differential Diagnosis

As mentioned above, Parkinson's disease is diagnosed clinically as there is no specific diagnostic test available. A confirmed diagnosis of PD is only possible through post-mortem examination, which confirms the degeneration of the substantia nigra and the presence of Lewy body pathology. In certain circumstances, both structural and dopaminergic imaging can be useful; however, PD can mimic other disorders due to the diversity and heterogeneity of its presentation and disease progression. Certain conditions can resemble idiopathic PD, and sometimes an accurate diagnosis may not be immediately apparent. Nonetheless, by paying attention to specific features during the patient's medical history and examination, it is possible to distinguish between these conditions.

#### 6.2.1. Dementia with Lewy body

Dementia with Lewy bodies (DLB) is a common form of dementia, second only to Alzheimer's disease. It involves progressive cognitive decline, fluctuating attention levels, visual hallucinations, REM sleep behavior disorder, and Parkinsonism. Both PD and DLB share the characteristic of Lewy bodies, which are primarily composed of α-synuclein. There is a considerable clinical and pathological overlap between the two conditions. Diagnostic criteria specify that if cognitive symptoms appear before or within one year of Parkinsonism, the diagnosis is DLB. However, if cognitive impairment develops in the context of established PD, the diagnosis is Parkinson's disease dementia [49].

#### 6.2.2. Progressive supranuclear palsy

Progressive supranuclear palsy, also known as PSP, is a tauopathy and a form of neurodegenerative disorder that can typically be distinguished from Parkinson's disease by its unique symptoms. PSP generally manifests as walking difficulties, shakiness, falls, and visual symptoms such as dry eyes and hazy vision in individuals in their 60s. PSP Parkinsonism, which differs from PD in that it affects the axial musculature, is symmetrical and does not react well to levodopa medication. In contrast to PD patients, who frequently exhibit retrocollis, PSP patients generally have an upright posture. Early and severe postural instability in PSP can cause repeated backward falls, especially when turning. A clinical examination, called the pull test, is used to evaluate postural instability. Moreover, freezing of gait can be a prominent symptom in PSP and does not respond well to levodopa therapy [50].

#### 6.2.3. Multiple system atrophy

A rare kind of neurodegenerative disease, called multiple system atrophy (MSA), is characterized by a variety of clinical signs and symptoms, such as Parkinsonism, cerebellar ataxia, autonomic failure, urogenital dysfunction, and corticospinal involvement. Contrary to Parkinson's disease, cognition typically remains intact. There are two subtypes of MSA: predominant parkinsonism (MSA-P) and predominant cerebellar ataxia (MSA-C). MSA-related Parkinsonism advances quickly and typically does not respond to levodopa medication. A traditional pill-rolling tremor is a rare sign, with bradykinesia and rigidity often predominating. Early on, it can be difficult to distinguish between MSA and PD because the symptoms are frequently asymmetrical and some individuals respond well to levodopa medication at first. However, patients with MSA treated with levodopa may exhibit atypical dyskinesias, such as prolonged facial dystonia or torticollis [51].

#### 6.2.4. Corticobasal syndrome

CBS is a disorder characterized by the degeneration of corticobasal structures, which can present in various ways. It has a classical presentation, as well as three other clinical phenotypes that are associated with corticobasal degeneration pathology. The diagnosis of CBS is primarily based on its motor features, which include asymmetric limb rigidity or akinesia, dystonia, and myoclonus. Parkinsonism may also be present, but is usually resistant to levodopa therapy and may involve the axial musculature. If present, tremors are not the typical rest tremors seen in PD [52].

## 7. Classical and novel therapeutic approaches to Parkinson's disease

The replacement of dopamine through the systemic administration of the amino acid L-DOPA was a significant advancement in Parkinson's disease treatment that occurred over 50 years ago and was a revolutionary breakthrough. However, current therapies for PD treat only the symptoms of the disease. There are several potential targets for pharmacological intervention aimed at modifying Parkinson's disease, including addressing neuroinflammation, mitochondrial dysfunction, oxidative stress, calcium channel activity, LRRK2 kinase activity, α-synuclein accumulation, aggregation, and cell-to-cell transmission. Additionally, immunotherapy techniques can be utilized in this context. Furthermore, surgical interventions such as targeted gene therapy, cell transplantation, and deep brain stimulation of subthalamic nuclei, can be considered potential approaches.

It is important to distinguish between classical treatments, which are well-established and commonly used in clinical practice such as L-DOPA, catechol-O-methyltransferase inhibitors, monoamine oxidase inhibitors, dopamine agonists, amantadine, and deep brain stimulation, novel treatments, which are still in the experimental or research stage, show promise as potential future therapeutic approaches such as stem cell therapy, graft assisted neural reconstruction, novel targets for disease modification, neurotrophic factors, and cannabinoids. Refer to Figure 8 and Table 1 for a concise overview of these classical and novel treatments for Parkinson's disease.

### 7.1. L-DOPA

The discovery of L-3,4-dihydroxyphenylalanine (L-DOPA) as a key therapeutic option for PD has revolutionized the management of this debilitating disease. L-DOPA, an amino acid precursor of dopamine that can cross the blood-brain barrier, is changed into dopamine in the presynaptic terminals of dopaminergic neurons by the enzyme aromatic amino acid decarboxylase (AADC). L-DOPA is considered to be the most successful symptomatic treatment for Parkinson's disease, and its use has greatly enhanced the quality of life for millions of patients throughout the world. It has been demonstrated to reduce tremors, stiffness, and bradykinesia and is suggested for the treatment of both early and severe stages of PD. L-DOPA can help lessen non-motor symptoms like anxiety, apathy, and despair. Although its curative effects can endure for years, the dosage may need to be changed if the disease worsens. To increase the effectiveness of L-DOPA, medications such as carbidopa or benserazide are commonly used to prevent peripheral metabolism of dopamine by inhibiting the enzyme AADC; this reduces the peripheral actions of dopamine (mainly the stimulation of the CTZ in the area postrema and resulting nausea and vomiting) and improves the bioavailability of L-DOPA in the CNS, allowing more L-DOPA to reach the brain, where it can be converted to dopamine to help alleviate the symptoms of Parkinson's disease.

The long-term use of L-DOPA in PD is associated with the development of motor complications, including motor fluctuations and dyskinesias, which can significantly reduce the quality of life of patients. The reasons behind the development of dyskinesias with long-term L-DOPA use are not fully understood. Both presynaptic and postsynaptic mechanisms are involved in this phenomenon, which results in the non-physiological pulsatile stimulation of striatal dopamine receptors and leads to various maladaptive neuronal responses [53]. A collateral problem is that L-DOPA's duration of action decreases, because as the population of dopaminergic neurons decreases, it is no more effectively taken up by the neurons, transformed in dopamine, and gradually released.

In order to address the problem of dyskinesias resulting from chronic L-DOPA replacement, researchers have been developing novel sustained-release formulations of L-DOPA, as well as methods for continuous delivery. These sustained-release formulations and delivery methods can be administered through either percutaneous endoscopic gastro-jejunostomy tubes or mini-pumps, and are designed to provide a more consistent and controlled release of L-DOPA over an extended period of time, thereby reducing the likelihood of non-physiological pulsatile striatal dopamine receptor stimulation and maladaptive neuronal responses [54].

### 7.2. Catechol- O -methyltransferase inhibitors

Catechol-O-methyltransferase (COMT) inhibitors are drugs used to treat Parkinson's disease. They work by blocking the action of an enzyme called COMT, which is responsible for breaking down dopamine in the brain. By blocking the breakdown of dopamine, COMT inhibitors increase the amount of dopamine available in the brain, which helps to improve the motor symptoms associated with Parkinson's disease.

There are two types of COMT inhibitors available: entacapone and tolcapone. Entacapone is a reversible inhibitor of COMT, while tolcapone is a reversible inhibitor of both COMT and another enzyme called COMT-B.

When used in combination with levodopa, COMT inhibitors have been shown to reduce the amount of “off” time experienced by patients with advanced Parkinson's disease, as well as increase the amount of “on” time and reduce the required dose of levodopa. Additionally, these inhibitors can improve the quality of life of Parkinson's disease patients by reducing motor fluctuations [55].

### 7.3. Monoamine oxidase inhibitors

Synaptically released dopamine is cleared from the brain via several mechanisms, including oxidation by monoamine oxidase type B in glial cells. This is one of the major mechanisms of dopamine clearance, along with presynaptic reuptake via the dopamine transporter. By inhibiting MAO, these drugs increase the availability of dopamine and other neurotransmitters in the brain, which can help improve the motor symptoms of PD. Non-selective MAO inhibitors, such as phenelzine and tranylcypromine, inhibit both MAO-A and MAO-B, while selective MAO inhibitors, such as selegiline and rasagiline, selectively inhibit MAO-B. Selective MAO inhibitors are preferred over non-selective MAO inhibitors due to their superior safety profile and diminished side effects. Both selegiline and rasagiline have FDA approval for the treatment of Parkinson's disease. Rasagiline can be used as either a monotherapy in the early stages of the disease or as an adjunct therapy in the later stages, whereas selegiline is commonly used as an adjunct therapy to levodopa in the later stages of the condition. Selegiline and rasagiline have both been proven in studies to reduce motor symptoms and postpone the need for levodopa medication [56].

### 7.4. Dopamine agonist

Two kinds of dopamine receptors, the D1 and D2 receptor families, mediate the effects of dopamine on medium-spiny neurons in the striatum. The D2 receptor family is the primary target of dopamine receptor agonists, which directly activate dopamine receptors. Bromocriptine, the first dopamine receptor agonist, was originally made available in the 1970s, and has since grown in importance as a treatment for Parkinson's disease's motor symptoms. Ergot alkaloid derivatives, which also stimulate 5-hydroxytryptamine (5-HT) receptors, including the 5-HT2B subtype, were the first dopamine receptor agonists. Unfortunately, these medications were linked to major adverse effects like cardiac valvular fibrosis and pleuropulmonary, which generated significant safety concerns. Modern dopamine receptor agonists lack this effect because they are non-ergoline medicines such as ropinirole, pramipexole, and rotigotine [57]. Longer-acting agonists may help at late stages when the action of L-DOPA becomes short and erratic. Extended-release medications have some potential benefits over immediate-release formulations, including a more consistent and stable level of dopaminergic activity with steadier plasma levels, greater tolerability, increased patient compliance with a simpler once-daily dosing regimen, and easier dose titration. A recent study conducted on early Parkinson's disease patients found that ropinirole-prolonged release was as effective and well tolerated as immediate-release ropinirole, but with significantly greater patient compliance. This study was conducted as a randomized, double-blind, non-inferiority, crossover study [58].

### 7.5. Amantadine

Amantadine is primarily used as an add-on treatment to levodopa therapy in patients with advanced PD who experience fluctuations in response to levodopa. It has also been used as a monotherapy in early PD and as an adjunct therapy in atypical Parkinsonian disorders.

Studies have shown that amantadine can reduce the duration of 'off' periods, increase “on” time without troublesome dyskinesias, and improve motor symptoms in patients with advanced PD. In addition, it has been shown to improve dyskinesias, which are often associated with long-term levodopa therapy [57].

### 7.6. Deep brain stimulation

For patients with advanced Parkinson's disease who continue to develop motor problems despite receiving the best possible medical care, deep brain stimulation surgery is a possibility. To target certain brain regions for electrical stimulation and regulation during DBS, an electrode is implanted in specific brain regions and high-frequency electrical stimulation (100–200 Hz) is delivered to mimic the effect of a lesion without destroying brain tissue.

**Figure 7.**
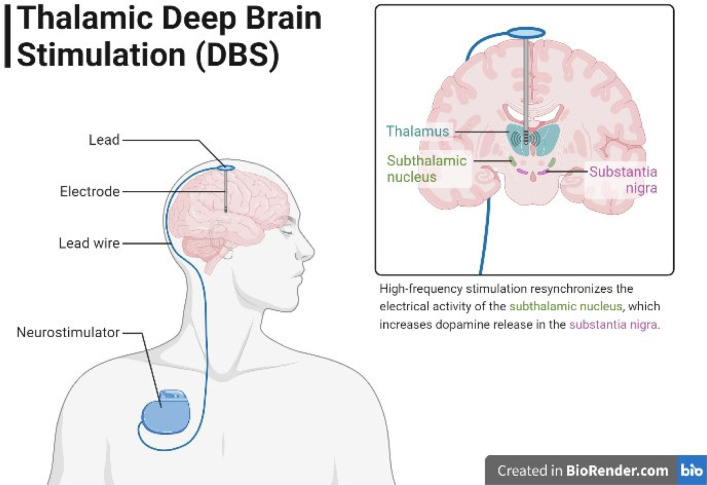
Illustration depicting the concept of thalamic deep brain stimulation (DBS). A coronal section of a brain with the thalamus highlighted shows the placement of the DBS lead within the thalamic region. Electrical signals are represented in blue traveling from the implanted pulse generator to the thalamus, symbolizing the delivery of stimulation. This high-frequency stimulation synchronizes the electrical activity of the subthalamic nucleus, leading to an elevation in dopamine release within the substantia nigra. Thalamic DBS is a neurosurgical procedure used for treating various neurological disorders including Parkinson's disease.

The subthalamic nucleus (STN) is the most often used target for DBS in PD. In patients with advanced PD, STN-DBS has been demonstrated to improve motor function, lessen motor problems, and increase quality of life. See Figure 7. However, DBS is ineffective at treating PD's non-motor symptoms and does not affect the underlying disease process [59].

DBS is normally only used on patients who have either underperformed during medicinal therapy or who have had serious adverse drug reactions. Patients are thoroughly evaluated before having DBS to make sure they are good candidates for the operation. This entails determining the prevalence of cognitive and mental comorbidities, determining the degree of motor impairment, and conducting neuroimaging studies to validate the existence of distinctive brain abnormalities linked to PD. Patients with dementia, acute psychosis, and major depression are not eligible for DBS, and these are considered exclusion criteria. Patients with young-onset Parkinson's disease are more likely to meet the inclusion criteria for DBS and are overrepresented among those who undergo the procedure. While older age is not necessarily an exclusion criterion for DBS, surgical complications are more common in this age group [59].

**Figure 8.**
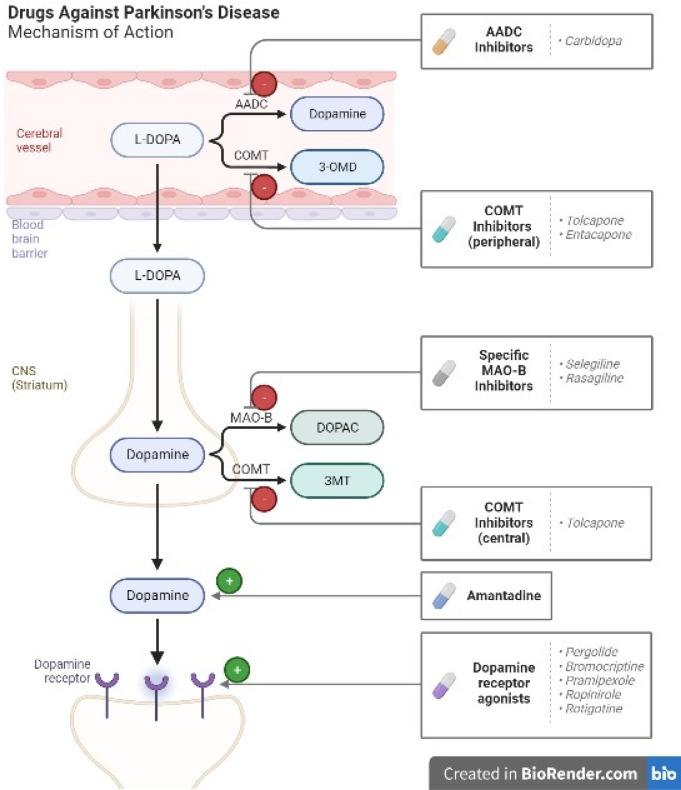
The figure illustrates the mechanism of action of drugs used for Parkinson's disease management. The diagram showcases the key components involved in the process. For a detailed summary of these treatments, refer to Table 1.

### 7.7. Stem cell therapy

In animal models of Parkinson's disease, experimental stem cell therapy has advanced significantly over the last 10–15 years. These days, two types of human pluripotent stem cells—human embryonic stem cells and human induced pluripotent stem cells—can be used to create dopaminergic neurons with midbrain characteristics. Recent research has demonstrated that they endure grafting into animals, develop into brain-innervating axons, and enhance functional recovery from lesion-induced deficits. As regulatory requirements for biological products rise, current research is concentrated on finding solutions to problems with scaling up cell manufacturing, ensuring safety, and achieving those requirements [60].

### 7.8. Graft-assisted neural reconstruction

A possible novel PD treatment called graft-assisted neural reconstruction (GANR) involves replacing the substantia nigra's deteriorated cells with healthy dopamine-producing cells in the brain. In this method, skin cells or other cells from the patient's own body are reprogrammed to become dopamine-producing neurons. The brain is then given these neurons so they may integrate into the existing neural network and start producing dopamine there [61].

Animal studies have indicated the possibility of GANR as a Parkinson's disease treatment, where it has been proven to enhance motor performance and lessen the need for dopamine replacement therapy. However, the clinical application of GANR is still in its early stages, and more research is needed to optimize the technique and assess its long-term safety and effectiveness.

A few of the issues associated with GANR include finding the optimum cell source for transplantation, maintaining the transplanted cells' survival and integration, and lowering the risk of immunological rejection. The transplanted cells could result in tumors or have other unfavorable outcomes; however, this risk can be reduced with appropriate monitoring and follow-up.

Notwithstanding these difficulties, GANR offers a lot of promise as a treatment for Parkinson's disease, since it may permanently restore dopamine production and enhance motor function. The goal of ongoing research in this field is to improve the procedure, discover the best cell sources for transplantation, and evaluate the long-term safety and efficacy of GANR in clinical studies [62].

### 7.9. Novel targets for disease modification

The molecular pathogenesis of Parkinson's disease is thought to be primarily driven by the oligomerization and fibrillar aggregation of pathological aSYN species, as well as their potential cell-to-cell transmission. This has brought the multimerization and extracellular and intracellular handling of this protein into sharp focus as potential targets for novel therapies. Two immunological strategies are being developed clinically right now. First, active immunization involves a novel vaccine. In a transgenic mouse model of Parkinson's disease, active immunization with a novel vaccine (AFFITOPE, AFFiRiS) containing short peptides homologous to aSYN conjugated to a carrier caused the formation of antibodies specifically directed against the carboxy terminus of human aSYN, cleared aSYN aggregates, and decreased neuropathology. Second, passive immunization can occur monoclonal antibodies against aSYN [63]. Other methods of targeting aSYN concentrate on extracellular aSYN binding sites, inhibitors of aSYN aggregation, or promoters of aSYN clearance via the LAS, although they have not yet advanced to the stage of clinical development. Other experimental methods, however, focus on various targets, such as the agonist of the glucagon-like peptide 1 receptor (exenatide), the urate precursor (inosine), the GBA chaperone (ambroxol), or the calcium channel antagonist (isradipine), some of which are currently being tested in Parkinson's disease clinical trials.

### 7.10. Neurotrophic factors

Neurotrophic factors, including glial cell line-derived neurotrophic factor (GDNF), have been shown to have positive effects on dopaminergic neurons in pre-clinical models of Parkinson's disease. This has generated significant interest in the development of neuroprotective therapies based on these factors. Studies that have used intra-putamen infusion of GDNF in an open-label format have observed improvements in motor UPDRS scores, as well as some indications of restoration of the nigrostriatal pathway, both pathologically and through imaging. Although studies involving GDNF have produced inconsistent results so far, this experimental approach is still considered to be exciting and there is ongoing interest in it [64].

### 7.11. Cannabinoids

There is limited clinical trial evidence on the effects of cannabinoids on Parkinson's disease symptoms. Most trials have either included fewer than 20 patients or have not been controlled. However, several uncontrolled observational studies have reported improvement in both motor and non-motor symptoms after taking cannabinoids, including reduced rest tremor, bradykinesia, rigidity, and levodopa-induced dyskinesia (LID), as well as improved pain, depression, psychosis, hallucinations, orientation, symptoms of RBD, and sleep quality [65]. Although no data from randomized clinical trials (RCT) currently exists on the use of cannabinoids to treat non-motor symptoms in PD, RCTs that have evaluated the impact of cannabinoids on quality of life in Parkinson's disease patients have reported either an improvement or no benefit [66]. One such drug is Nabilone. It is a derivative of tetrahydrocannabinol (THC) - the psychoactive component of cannabis - but it is not obtained directly from the cannabis plant. It behaves as a partial agonist on both CB1 and CB2 receptors in humans, thereby producing effects similar to THC but with fewer side effects and less likelihood of causing euphoria. Nabilone is already available in the market and is generally considered safe and well-tolerated. Endogenous and exogenous cannabinoids, including nabilone, may have potential therapeutic benefits for improving sleep and relieving pain and mood disorders in PD patients through their effects on various neurotransmitter systems, including monoaminergic, GABAergic, glutamatergic, and opioid signaling. Although observational studies suggest a positive impact of cannabinoids on non-motor symptoms in PD patients, further research is needed to confirm these effects in controlled trials and to determine their potential for broader use in managing non-motor symptoms in PD patients [67].

**Table 1.** Classical and Novel Therapeutic Approaches to Parkinson's Disease with important points to be aware of.

**Treatment**	**Key Features**	**References**
L-DOPA	Systemic administration of L-DOPA, a precursor of dopamine, is the most successful symptomatic treatment for PD, alleviating motor and non-motor symptoms. Long-term use may lead to motor complications. Researchers are developing sustained-release formulations to address dyskinesias.	[53],[54]
Catechol-O-methyltransferase inhibitors (COMT inhibitors)	These drugs block the enzyme COMT, increasing dopamine levels in the brain and reducing motor fluctuations when used with levodopa.	[55]
Monoamine oxidase inhibitors (MAO inhibitors)	MAO inhibitors increase dopamine availability by blocking its clearance from the brain, improving motor symptoms. Selective MAO-B inhibitors, such as selegiline and rasagiline, are preferred due to their safety profile.	[56]
Dopamine agonists	Dopamine receptor agonists stimulate dopamine receptors and are used to treat motor symptoms of PD. Non-ergoline agonists like Ropinirole, Pramipexole, and Rotigotine have replaced older medications with fewer side effects.	[57],[58]
Amantadine	Used as an add-on treatment to levodopa, amantadine reduces 'off' periods and dyskinesias in advanced PD patients.	[57]
Deep brain stimulation (DBS)	DBS involves implanting electrodes to stimulate specific brain regions, typically the subthalamic nucleus, to improve motor function in advanced PD cases.	[59]
Stem cell therapy	Experimental stem cell therapy aims to replace dopamine-producing cells in the brain using human pluripotent stem cells. Currently in preclinical stages.	[60]
Graft-assisted neural reconstruction (GANR)	GANR involves replacing deteriorated substantia nigra cells with reprogrammed dopamine-producing neurons from the patient's own body. Under early-stage clinical evaluation.	[61],[62]
Novel targets for disease modification	Research focuses on modifying the underlying disease process, targeting aSYN species, extracellular/intracellular handling, and other potential novel therapies.	[63]
Neurotrophic factors	Glial cell line-derived neurotrophic factor (GDNF) has shown positive effects on dopaminergic neurons in preclinical models. Ongoing interest in its development.	[64]
Cannabinoids	Limited clinical trial evidence exists, but cannabinoids have shown potential in improving motor and non-motor symptoms in observational studies. Requires further research.	[65]–[67],[73]

## 8. Conclusion

Dopamine plays a crucial role in the pathophysiology of Parkinson's disease, which by itself, is a complex, multifactorial disease. The vulnerability of dopaminergic neurons in the substantia nigra pars compacta in Parkinson's disease remains a major topic of research in the field. As mentioned in this review, while the loss of SNpc dopaminergic neurons causes the primary motor symptoms of PD, the pathology is not solely restricted to these neurons. Two primary explanations for the destruction of SNpc dopaminergic neurons are the presence of Lewy pathology, which involves protein aggregates rich in fibrillary forms of alpha-synuclein, and mitochondrial malfunction. The susceptibility of SNpc DA neurons to damage from these sources is not absolute, and its cause is not completely clear, although the oxidative action of DA and its metabolites, the sensitivity of aSYN to oxidation, and its action in stabilizing the synaptic vesicle likely contribute. The precise relationship between aSYN pathology and cell death remains uncertain, although several possible mechanisms have been suggested. It is important to further dissect the role of aSYN in regulating dopaminergic neurotransmission and in defining the characteristics of dopaminergic neurons, at a cellular and molecular level, that lead to their preferential neurodegeneration in PD. Understanding the mechanisms underlying the vulnerability of SNpc dopaminergic neurons in PD remains critical for developing new therapeutic approaches to halt or slow the progression of this debilitating disease.

The current therapies for Parkinson's disease are based on the replacement of dopamine and the modulation of dopamine pathways. Levodopa remains the most effective therapy, providing symptomatic relief to the motor symptoms of Parkinson's disease. However, long-term use of levodopa can lead to motor complications, and the development of non-motor symptoms remains a challenge. Other therapies, such as COMT inhibitors, MAO inhibitors, dopamine agonists, and amantadine, are used in combination with levodopa or as monotherapy in the early stages of the disease. Although current treatments for Parkinson's disease are often effective in improving motor function, they are associated with significant side effects. These side effects result from the way the medications deliver dopamine to extra-striatal regions and the variability in their absorption and transit across the blood-brain barrier. In addition, the continuous release of dopamine caused by these medications is non-physiological and can affect dopamine receptors within the basal ganglia. As a result, patients frequently experience cognitive problems, levodopa-induced dyskinesias (uncontrolled, involuntary movements), and on-off fluctuations (periods when the medication is effective, followed by periods when it is not). In fact, based on data from an ongoing community-based incident study of Parkinson's disease, it is estimated that 46% of patients will experience cognitive problems, 56% will experience levodopa-induced dyskinesias, and 100% will experience on-off fluctuations within 10 years of diagnosis. These side effects, coupled with some of the neuropsychiatric features of Parkinson's disease, can have a significant impact on the quality of life of patients as the disease progresses.

Furthermore, many of the features of Parkinson's disease have a mainly non-dopaminergic basis. These features result from the neurodegeneration of other areas in the central nervous system as well as the enteric and autonomic nervous systems. Such features include cognitive impairment and autonomic dysfunction, which can have a greater impact on a patient's quality of life than motor symptoms. Unfortunately, treatment options for these non-motor features of Parkinson's disease are limited, leaving patients with few options for managing these symptoms.

Thus, there is a pressing need for improved therapies, including treatments that modify the progression of Parkinson's disease. However, developing such treatments is challenging due to several factors. First, relevant pre-clinical disease models are necessary to test potential therapies, but they can be difficult to develop. Second, there is a lack of reliable biomarkers for diagnosing Parkinson's disease and identifying the early stages of the disease before significant neuronal loss has occurred. Without these biomarkers, it is difficult to identify and treat Parkinson's disease in its early stages, when interventions are most likely to be effective. These challenges pose significant barriers to drug discovery and the development of disease-modifying treatments for Parkinson's disease.

Various invasive approaches are being explored to provide continuous local dopaminergic stimulation within the striatum. One option is cell replacement therapy, but clinical transplantation research has been halted after two separate sham-controlled trials failed to show the efficacy of fetal mesencephalic transplantations in PD [68]. Another approach involves intraputaminal implantation of human retinal pigment epithelial (RPE) cells, which produce levodopa and act as local levodopa “bioreactors” without making synaptic contact with the surrounding tissue. In a pilot study, six patients with advanced PD who received unilateral implantation of RPE cells had an average improvement of 48% in the off-period motor score after one year [69]. Another approach involves intrastriatal infusion of an adeno-associated viral vector containing the gene for human aromatic L-amino acid decarboxylase, which has been reported in a phase I safety trial. Transduced striatal neurons can convert levodopa to dopamine, potentially leading to a more stable intrastriatal dopamine release [70]. These therapies are still experimental and not yet ready for clinical practice, but they illustrate the importance of the concept of continuous dopaminergic stimulation in therapeutic research for PD.

The outcomes of certain clinical trials investigating drugs for possible disease-modifying effects in PD have not been successful. The causes for these results may be diverse, including the clinical and pathophysiological diversity of PD and the challenge in detecting pre-motor PD. Additionally, the absence of reliable and objective measures to assess the efficacy of drugs may also contribute to unfavorable results. However, the potential to address these challenges exists through the use of various cerebrospinal fluid biomarkers. aSYN being a key player in the molecular pathways associated with PD pathogenesis is a commonly used biomarker for evaluating drug efficacy in clinical trials. In summary, CSF biomarkers show potential as effective outcome measures for clinical trials testing disease-modifying treatments for PD. However, diagnostic and prognostic biomarkers for PD have not been fully validated yet and are primarily used for research purposes at the moment.
